# A new highly active La_2_O_3_–CuO–MgO catalyst for the synthesis of cumyl peroxide by catalytic oxidation†

**DOI:** 10.1039/d1ra00176k

**Published:** 2021-03-30

**Authors:** HanShuang Liu, KaiJun Wang, XiaoYan Cao, JiaXin Su, Zhenggui Gu

**Affiliations:** Jiangsu Provincial Key Laboratory of Materials Cycling and Pollution Control, Nanjing Normal Univesity Nanjing Jiangsu 210023 China 07160@njnu.edu.cn

## Abstract

In this study, different magnesium, copper, lanthanide single metal, and composite multimetal oxide catalysts were prepared *via* the coprecipitation route for the aerobic oxidation of cumene into cumene hydroperoxide. All catalysts were characterized using several analytical techniques, including XRD, SEM, EDS, FT-IR, BET, CO_2_-TPD, XPS, and TG-DTG. La_2_O_3_–CuO–MgO shows higher oxidation activity and yield than other catalysts. The results of XRD and SEM studies show that the copper and magnesium particles in the catalyst are smaller in size and have a distribution over a larger area due to the introduction of the lanthanum element. The CO_2_-TPD results confirmed that the catalyst has more alkali density and alkali strength, which can excite active sites and prevent the decomposition of cumene hydroperoxide. XPS results show that due to the promotional effect of La_2_O_3_, there are more lattice and active oxygen species in the catalyst, which can effectively utilize the lattice defects under the strong interaction between metal oxides for rapid adsorption and activation, thus improving the oxidation performance. Besides, La_2_O_3_–CuO–MgO exhibits good stability and crystalline structure due to its high oxygen mobility inhibiting coking during the cycle stability test. Finally, the possible reaction pathway and promotional mechanism on La_2_O_3_–CuO–MgO in cumene oxidation are proposed. We expect this study to shed more light on the nature of the surface-active site(s) of La_2_O_3_–CuO–MgO catalyst for cumene oxidation and the development of heterogeneous catalysts with high activity in a wide range of applications.

## Abstract 2

The La_2_O_3_–CuO–MgO catalyst acts on the oxidation of cumene and shows excellent catalytic activity through the coordination of surface and interior.

## Graphical abstract

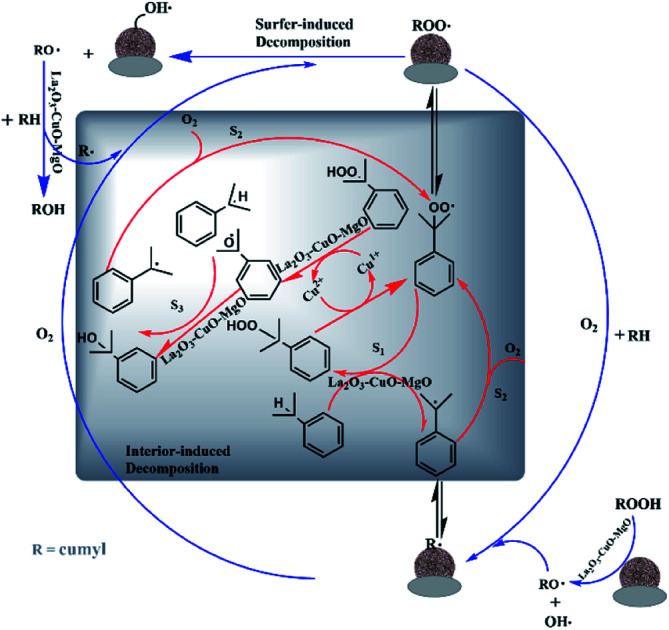

## Introduction

Currently, liquid-phase oxidation of hydrocarbons is having a profound impact on the industry and economy and can be regarded as the core of chemical synthesis. This reaction plays an important role in turning petroleum hydrocarbon raw materials into industrial chemicals, and in polymer and petrochemical industries.^1^ For example, the oxidation of cumene is one of the important way to produce cumene hydroperoxide (CHP), phenol, and acetone in industry.^2,3^ CHP is used in industry as an initiator for chain autoxidation and polymerization reactions, an accelerator for rubber vulcanization, an oxidant in fine chemicals, and in polymer synthesis.^4,5^ Phenol is an important raw material for phenol resins, perfumes, medicines, agricultural chemicals, and many other industries.^6^ At present, more than 90% of phenol production in the world is based on this route.^7^ The products or by-products of the whole process are of great industrial value and the chemical reaction involves extremely high atom utilization.

The liquid-phase oxidation of isopropyl-aromatic hydrocarbons proceeds according to a widely-known free radical chain mechanism. In most industrial production processes, cumene oxidation is typically carried out in the liquid phase, using O_2_ (or air) as an oxidant in the absence of any catalyst (auto-oxidation process) and with small amounts of CHP or other substances added as initiators. Cumene conversion is usually kept low to maximize the selectivity toward CHP and minimize side products resulting from CHP decomposition, mainly 2-phenyl-2-propanol (cumyl alcohol, PP) and aceto-phenone (AP).^3^ Despite these precautions, the productivity of the auto-oxidation process is generally below the desired values. Therefore, in order to solve this problem, it is very necessary to develop highly active and reusable homogeneous and heterogeneous catalysts for cumene oxidation to increase the productivity of CHP. Recent research on the liquid-phase oxidation of cumene-involved metal–organic frameworks (MOFs),^8,9^ Salenalen-type complexes,^10^*N*-hydroxyphthalimide (NHPI),^11^ ionic liquids,^12^ and metal oxides as important research components. Many homogeneous catalysts can improve CHP yield under mild conditions, but they have become stumbling blocks in the process of catalyst preparation, product purification and recovery. When used in the actual production process, it is difficult to achieve the expected results with above catalysts. Heterogeneous catalysts are widely used in the synthesis of chemical products and have the advantages of simple preparation, easy recycling, and long catalytic life. It is the main trend in the development of the chemical industry and one of the important way to realize green chemical processes.

In past decades, many heterogeneous composite metal oxide catalysts were investigated for the oxidation of cumene. In experimental studies on oxidation processes, many commonly-used transition metal catalysts, such as Cu(i)/Cu(ii), Co(ii)/(iii), Fe(ii)/(iii) and Mn(ii)/(iii), are typically investigated as oxides,^13^ complexes^14^ or loaded catalysts.^15^ Hsu *et al.*^16^ studied heterogeneous catalysis on transition metal oxides from the fourth period for cumene oxidation, and found that these metal oxides have both chain initiation and reaction acceleration capabilities. Finally, 6.85% IPB conversion and 99% CHP selectivity were obtained at 353 K. For all the above-mentioned catalytic systems, copper compounds were excellent catalysts not only with regard to the reaction activity but also with regard to the CHP selectivity.^17,18^ Marella R. K. *et al.*^19^ found that the transition metal salt mixed with alkali metal oxides/alkaline earth metal compounds showed an obvious improvement in the catalytic activity. MgO is often chosen as a support to enhance the specific surface area of the catalyst.^20^ Based on infrared studies reported in the literature, the hydrogen atom on the isopropyl tertiary carbon of cumene chemically adsorbs on the lattice oxygen with a negative basic center on the MgO surface, thereby weakening the C–H bond on the isopropyl tertiary carbon and facilitating the reaction. Cho *et al.*^21^ found that C–H bonds were activated easily by Fe/MgO. V. V. Costa *et al.*^20^ investigated the liquid phase oxidation of various alcohols catalyzed by Au/MgO with molecular oxygen as an oxidant.

However, none of the proposed catalysts were widely introduced into the industry. Better suitability for industrial production, boosting the overall activity and selectively obtaining the desired products are significant challenges for cumene oxidation. There are few reports on the catalytic performance of multi-metal oxides for cumene oxidation. Therefore, based on the above-mentioned study on the oxidation of cumene with binary heterogeneous solid bases, this work was to continue exploration on the effects of rare earth oxide doping on the catalyst structure and oxidation performance. Some studies show that rare earth elements have become important doping elements in various catalytic materials in recent years due to their special electronic structure.^22–25^ The catalytic initiative and stability of the mixed metal oxides can be advanced by synergy with different metal ions.^26,27^ La is a typical rare earth metal with good oxygen storage and release capabilities, which can stabilize the structure of alkaline earth metal oxides, improving the dispersion of active constituents^24^ and reduce the apparent activation energy of the catalyst. Therefore, we report a high-activity La_2_O_3_–CuO–MgO ternary composite solid base catalyst and evaluate its excellent activity in the catalytic reaction of cumene. The material can be easily prepared by the coprecipitation method, and the strong oxidation capability of the prepared material is strongly proved by sufficient characterization. Rich lattice oxygen and active oxygen make this catalytic material have a long life. More importantly, this work discusses the mechanism of promoting the free radical reaction of cumene and could provide some directions and perspectives on heterogeneous catalytic oxidation of hydrocarbons.

## Results and discussion

### Structural and morphology characterization

#### XRD measurements

**Fig. 1:**
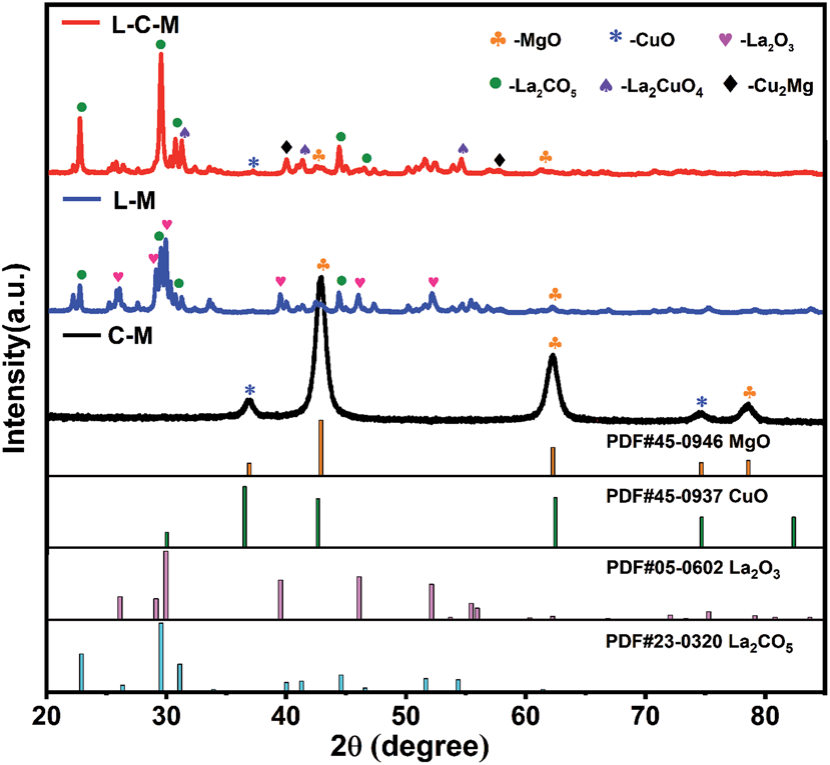
The XRD profiles of the C–M, L–M and L–C–M.

The XRD patterns of C–M, L–M and L–C–M catalyst samples are displayed in Fig. 1. The characteristic reflection of (200), (220), and (222) at 42.91°, 62.28°, and 78.59°, respectively, are assigned to the MgO phase. Two peaks at 36.49° and 74.67° can be recognized for (211) and (220) crystal planes, respectively, of CuO.^28^ The (100), (002), (101), (102), (110) and (103) at 26.11, 29.13°, 29.96°, 39.53°, 46.08° and 52.13° diffractions of the crystalline La_2_O_3_ phase are observed.^29,30^ The diffraction peaks of La_2_CO_5_ at 22.84°, 29.55°, 30.82°, 44.51° and 46.56° can be indexed as (011), (013), (110), (020), and (022) planes,^31^ respectively. For the multicomponent phase analysis, diffraction peaks of (113) and (311) planes of La_2_CuO_4_ appear at 31.12° and 54.73°, and diffraction peaks of (311) and (331) crystal planes of Cu_2_Mg appear at 39.28° and 57.17°, respectively.

The peak intensity and particle size of the MgO and CuO phases obviously decreased with the introduction of La. The results indicate that La_2_O_3_ can effectively improve the dispersion of MgO and CuO phases and may form a new compound of MgO and CuO in the medium or noncrystalline form. The observed increase in the distribution of those phases can be preliminarily assigned to the fact that the ion radius (0.106 nm) of La^3+^ is much larger than that of Mg^2+^ and Cu^2+^ (0.072 nm and 0.073 nm, respectively). As a result, MgO and CuO microcrystals are covered by La_2_O_3_ films, which block the adhesion of particles, limit their grain growth during heat treatment, or cause CuO to dissolve in the lattice of the solid solution of MgO, which forms CuO–MgO. Furthermore, it was observed that some La_2_O_3_ was dissolved in CuO lattice, thanks to the solid–solid interplay between La_2_O_3_ and CuO, which prompts the constitution of lanthanum cuprate (La_2_CuO_4_).^24^ In the diffraction pattern of L–C–M, no diffraction peak of the La_2_O_3_ phase was found, indicating the existence of extremely subtle La_2_O_3_ particles, whose grain size became very small and lower than the diffraction detection limit of the X-ray instrument. Nonetheless, the absence of La_2_O_3_ as a single-phase may likewise be due to its dissolution in the lattice of MgO and CuO, resulting in the constitution of new complexes in a shapeless state or a badly crystalline state.^32^

#### Morphology and structures of catalysts

**Fig. 2:**
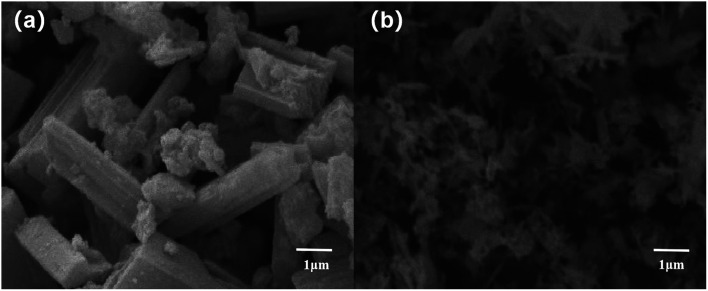
The SEM pictures of the (a) C–M, (b) L–C–M.

The morphology of C–M and L–C–M catalyst samples was analyzed using a scanning electron microscope, as shown in Fig. 2. It can be observed that C–M consists of relatively uniform aggregates of rod-like type^29,33^ of 2–3 μm of the width and 8–10 μm in length (Fig. 2a). These aggregates are formed by MgO platelets and CuO. After the C–M modification with La_2_O_3_, the particle size decreases from 3 to 0.1 and 10 to 1 μm of the width and length, respectively (Fig. 2b). Therefore, the addition of La_2_O_3_ reduces the particle size and improves the dispersion of the catalyst.

**Fig. 3:**
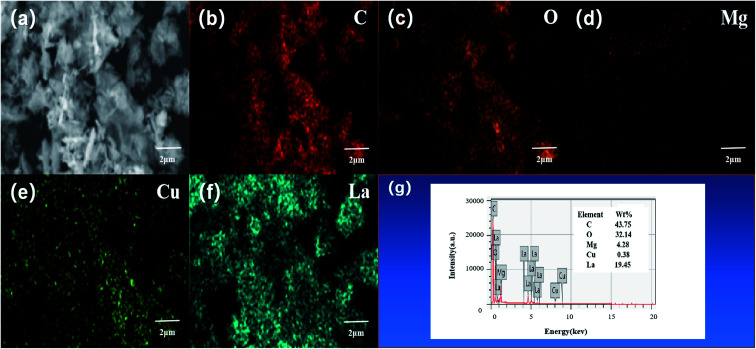
X-ray energy spectrum analysis results of L–C–M sample: (a) scanning electron microscope image; (b) carbon element distribution diagram; (c) oxygen element distribution diagram; (d) magnesium element distribution diagram; (e) copper element distribution; (f) lanthanum element distribution diagram; (g) catalyst sample test energy spectrum.

The element distribution and composition of the catalyst were further examined by EDS, as shown in Fig. 3g. Quantitative elemental analysis shows that the weight percentages of C, O, Mg, Cu, and La elements in the L–C–M catalyst sample were 43.75%, 32.14%, 4.28%, 0.38%, and 19.45%, respectively. The molar ratios of Cu/Mg and La/Mg elements maintain good consistency with that designed at the time of preparation. Fig. 3a–f are the mapping diagrams of C, O, Mg, Cu, and La elements, respectively, showing the distribution and density of each element on the sample surface. Compared with Fig. 2a, it can be observed that Mg, Cu, and La elements are uniformly distributed on the sample surface, indicating that the addition of La greatly improves the dispersion performance of the C–M catalyst. These changes make it easier for the metal oxide surface bind negatively charged lattice oxygen to the hydrogen atom on the isopropyl tertiary-carbon, weakening the bond energy between C–H bonds^34^ and increasing the catalytic activity of the catalyst. Besides, La_2_O_3_ itself has a good ability to store and release oxygen, which effectively enhances the catalytic life of the catalyst, which is consistent with XRD and O 1s XPS analysis.

#### FT-IR studies

**Fig. 4:**
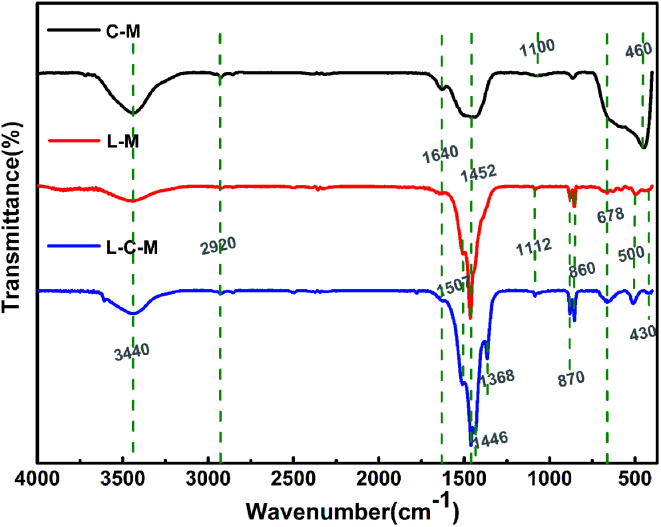
The FT-IR transmission spectra of the C–M, L–M, L–C–M.

Fourier transform infrared (FT-IR) spectroscopy is used essentially as an extra probe to demonstrate the existence of OH groups and also additional organic and inorganic chemical species. It not only proves the chemical changes but may also reveal the interplay between the two metals. The FTIR spectra in the range 4000–400 cm^−1^ of the C–M, L–M and L–C–M catalysts are shown in Fig. 4. For these three catalysts, the wide absorption zone of 3350–3750 cm^−1^ is related to the superposition of the hydroxyl stretch zone *ν*(OH_strs_) produced by water molecules between the metal hydroxyl group and the hydrogen bond layer. This indicates the presence of hydroxyl groups on the catalyst surface.^35^ The peak near 1640 cm^−1^ is the bending vibration of OH groups of physically adsorbed water. Another transmission band is about 2920 cm^−1^, corresponding to the OH tensile vibration of carboxylic acid and CH asymmetric tensile vibration. In the literature, the peaks at 1452 cm^−1^ and 860 cm^−1^ are ascribed toward the C–O extend the pattern and the crooking pattern of structural carbonate, individually. The bands observed in the low-frequency area may be ascribed to lattice vibration patterns of M–O–M (M = Mg, Cu, La) and carbonate vibrations.^36^ Powerful and pointed bands near 1507–1368 cm^−1^ are assigned to extending and twisting O–H vibrations of La(OH)_3_, with a general number of hydroxyl groups at the surface, which plays the role of basic sites. The absorption peak at 1100 cm^−1^ corresponds to magnesium propionate, and the band of CO^3−^ in the skeletal oscillation in propionates is situated around 1100–900 cm^−1^. The intensity of these bands decreases with the calcination of the catalyst, when the metal propionate decomposes fully to metal oxide. The frequency band below 800 cm^−1^ is mainly due to metal–oxygen vibrations.^37^ Another absorption band at about 678 cm^−1^ stands for the antisymmetric bending vibration of carbonate (*ν*_4_). When lanthanide was introduced, the absorption peaks at 870 and 860 cm^−1^ are the characteristic peaks at La_2_O_3_ formed by the decomposition of lanthanum propionate, and absorption peaks at 500, 460 and 430 cm^−1^ are assigned to CuO formed by the decomposition of copper propionate.^38^

#### BET analysis

**Fig. 5:**
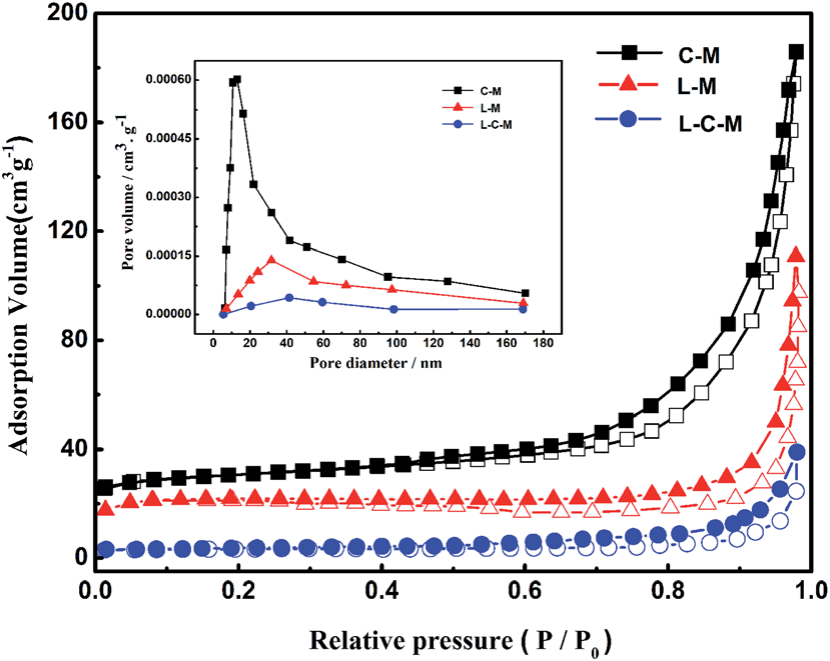
N_2_ adsorption–desorption isotherms and pore-size distribution curve (inset) of C–M, L–M, L–C–M.

Physical features regarding several catalysts are shown in Table 1, and the N_2_ adsorption–desorption isotherms of the obtained new catalysts are shown in Fig. 5. As is shown, the surface area and pore volume of catalysts decreased greatly with the addition of La_2_O_3_, perhaps due to the introduction of La_2_O_3_ particles into the pores of MgO or CuO, which caused a part of the pores of the catalyst to be covered or blocked, resulting in an order of magnitude reduction in the total pore volume. All catalysts showed Langmuir adsorption isotherms of type IV, which indicated that there was a large proportion of mesopores in the catalysts. According to IUPAC classification, the hysteresis in the isotherm for the catalyst is H3 type,^39^ and in L–M and L–C–M, pores are stacked in a slit shape. Its pore size distribution is more dispersed, which is more conducive to molecular diffusion and promotes the higher activity of the catalyst. Besides, a small amount of La_2_O_3_ in L–C–M diffuses to the CuO surface to form a CuO-based La_2_O_3_–CuO solid solution. Due to the powerful interaction between La and Cu, the bulk properties of the catalyst change, which also results in the decrease of particulate surface area and pore volume of catalyst.

**Table 1:** Physical properties of catalysts

Samples	BET surface area (m^2^ g^−1^)	Total pore volume (cm^3^ g^−1^)	Average pore diameter (nm)
C–M	48.98	0.28	22.87
L–M	20.84	0.18	30.15
L–C–M	4.15	0.06	43.58

Further, the BJH pore size distribution curve (inset), signifies that with the introduction of lanthanum element, the pore size peak intensity of the catalyst is significantly reduced, which further indicates the blockage of the catalyst pores, in agreement with XRD analysis outcomes.

#### Results of CO_2_-TPD analysis

**Fig. 6:**
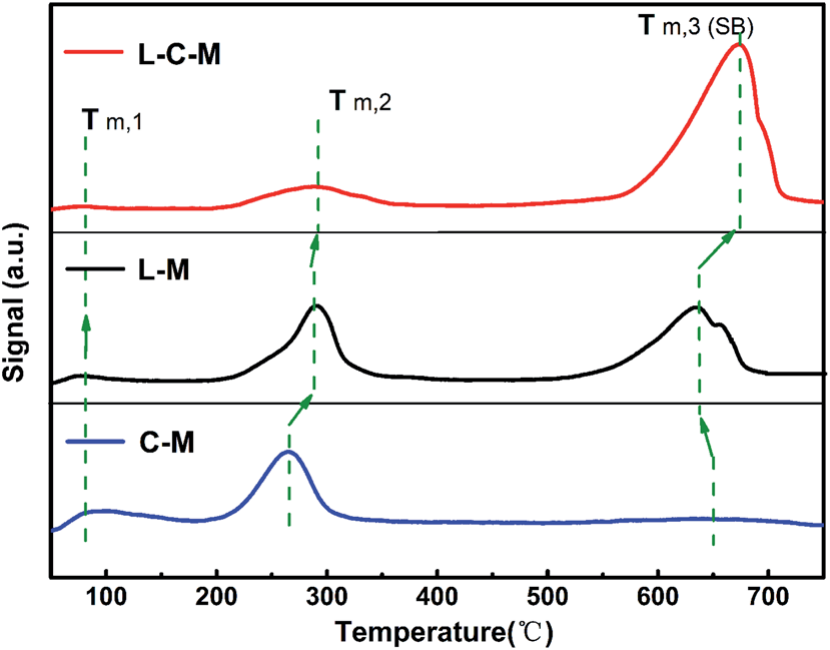
The CO_2_-TPD pictures of the C–M, L–M and L–C–M.

As for the as-formed C–M, L–M and L–C–M samples, the essential positions signify those commonly associated with O^2−^ ions on the surface.^40^ The surface alkalinity of catalyst samples was detected using the CO_2_-TPD technique.^29^ As exhibited in Fig. 6, all samples show three CO_2_ desorption peaks, among which the feeble Brønsted basic sites are concentrated in the 83–142 °C region, and the moderate Lewis basic sites are concentrated in the 196–355 °C region. The powerful Lewis basic sites are concentrated near 550–730 °C, which is connected to the surface OH^−^ groups and coordinated unsaturated O^2−^ ions. Nevertheless, the C–M sample presents a weak desorption peak at 670 °C, indicate that only a few strongly basic (SB) sites.

In light of the quantitative analysis on the denseness of basic sites on C–M, L–M and L–C–M samples (Table 2), the denseness of the total basic sites and SB sites are as follows: C–M < L–M < L–C–M. Previous CO_2_-TPD and FTIR studies^41,42^ have indicated that the hydroxy groups on the surface of La_2_O_3_ have basic sites. Combined with the results of XRD, due to the interaction between La_2_O_3_ and CuO/MgO, CO_2_ is adsorbed on the strong alkaline centers, that is, there are SB sites, which greatly increase the total alkali content.

**Table 2:** The alkaline test results of the C–M, L–M and L–C–M

Samples	Total basic sites	SB sites	
	Quantity (cm^3^ per g STP)	Temperature (°C)	Quantity (cm^3^ per g STP)
C–M	21.80	670.25	2.32
L–M	55.14	633.92	30.14
L–C–M	60.55	672.43	46.60

#### XPS results

**Fig. 7:**
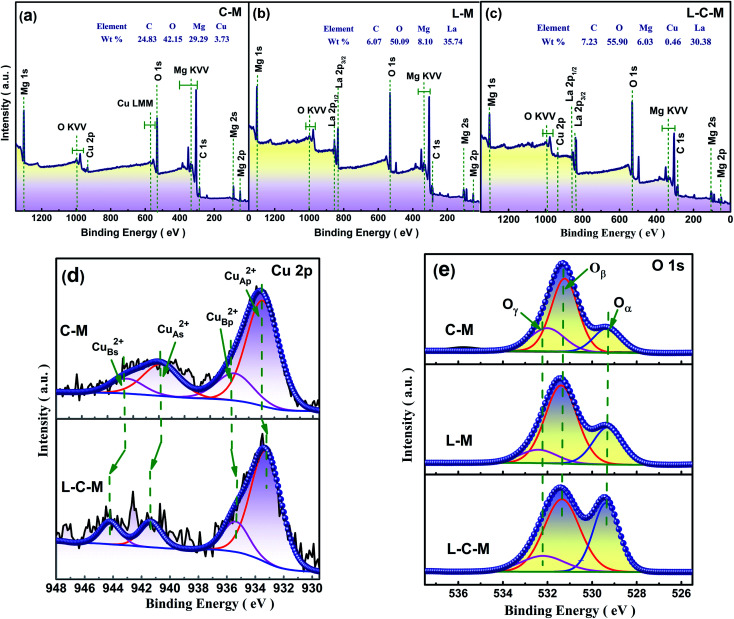
XPS survey spectrum of (a) C–M, (b) L–M and (c) L–C–M. High resolution XPS survey at (d) Cu 2p region, and (e) O 1s region.

As is known, the catalytic properties of catalysts are closely associated with their surface chemistry states. The surface component and electronic states of C–M, L–M and L–C–M were analyzed by X-ray photoelectron spectroscopy (XPS). The scanning spectra of the three samples show that there are corresponding C, O, Mg, Cu, and La elements on the surface, which is also very consistent with the elemental molar ratio analyzed using EDX (Fig. 7a–c). Table 3 shows the distribution characteristics of the corresponding element molar ratios of Cu 2p and O 1s. According to the overall trend shown in Fig. 7d, the main spectral peak (p peak) of Cu 2p in the catalyst doped with La tends to shift to low energy.^43^ Ordinarily, the binding energy (BE) of the primary Cu 2p_3/2_ peak (p peak) associated with Cu^2+^ type lies in the area of 933.28–935.66 eV, while its disturbed satellite thread (s peak) lies between 941.29–944.02 eV. There are obvious vibrational characteristics between 940–945 eV, which also excludes the possibility of Cu_2_O. The results indicate that Cu on the MgO surface of the prepared C–M and L–C–M catalysts exists in the form of Cu^2+^. Satellite peaks indicate that if sudden approximation (unipolar selection rule) and ligand field theory are used, Cu^2+^ compounds are caused by the unipolar charge transfer transition (ligand metal 3d). Since the 3d shell is filled, these satellite peaks cannot be observed in Cu^+^ compounds or metallic Cu.^44^ In fact, transition metal ions including unfilled 3d orbits are widely reported to display satellite peaks under the nuclear XP spectrum, which is owing to electronic vibration and satellite structure, that is, the number of peaks, intensity, and splitting reflecting chemical bond properties of transition metal ions.^45^ As for C–M and L–C–M samples, the peak at 933.25–933.33 eV (Cu_Ap_^2+^ peak) is attributed to the extremely scattered Cu^2+^ species within the metal oxide (Cu_A_^2+^), while the peak near 935.37–935.66 eV (Cu_Bp_^2+^ peak) may be related to the covalent Cu^2+^–O bond polarized by Mg^2+^ ions, thus reducing the efficient charge of Cu^2+^ species. Noticeably, the doping of rare earth metal La is not only beneficial for the appearance of the main peak Cu_Ap_^2+^ species but also beneficial for the formation of Cu_Bp_^2+^ species. This result can still be observed according to the data given in Table 3. Therefore, compared with the C–M catalyst, L–C–M not only has more highly dispersed Cu^2+^ species but also shows a strengthened interaction between Cu–Mg species. These results indicate the generation of lattice defects on the surface catalyst, resulting in more amorphous CuO–MgO solid solutions, which enhance the reaction activity of the catalyst. This is consistent with XRD, BET, and SEM analysis results.

**Table 3:** Surface atomic concentration of different elements in C–M, L–M and L–C–M catalysts using XPS results

Samples	Surfer element molar ratio/%						
	Cu_Ap_^2+^	Cu_Bp_^2+^	Cu_As_^2+^	Cu_Bs_^2+^	O_α_	O_β_	O_γ_
C–M	59.76	11.86	16.01	12.36	15.89	65.43	18.68
L–M	—	—	—	—	17.74	67.23	15.03
L–C–M	69.01	15.83	8.91	6.25	20.33	71.03	8.64

Taken into consideration that the source of alkaline sites is associated with the types of surface oxygen, XPS spectra of O 1s of samples were analyzed. As shown in Fig. 7e, the peak at 529.41 eV is attributed to the lattice oxygen (O_α_ and O^2−^) and the peak at 531.29 eV is attributed to the chemically adsorbed surface-active oxygen (O_β_, O^−^, and O_2_^2−^). The peak with the value of 532.23 eV may be related to hydroxyl groups or water molecules (O_γ_) adsorbed to the catalyst surface.^46^ It can be seen from Table 3 that due to the doping of La, the concentration of O_β_ is greatly increased, and O_β_ plays a vital role in the oxidation reaction and is consistent with great influence on the catalytic activity concerning the catalyst. The molar quotient of O_β_/(O_α_ + O_β_) on these catalysts follows the order, C–M < L–M < L–C–M. This may be because La_2_O_3_ and CuO/MgO in the catalyst are closely interlaced, which leads to defects, and molecular oxygen can be quickly absorbed and activated by vacancy defects in the catalyst. Therefore, active oxygen can be easily transferred to supplement the surface-active oxygen consumed in the oxidation process. Furthermore, it can be observed that the concentration of O_α_ is also increased significantly, which also indicates that the introduction of La can indeed promote the release of oxygen in the CuO/MgO lattice.^47^ According to the literature, the presence of lattice oxygen is beneficial to the selective oxidation of C–H compounds to produce aldehydes, ketones, peroxides, and other target products. The formation of strong alkaline sites on the catalyst surface can be attributed to the existence of lattice oxygen.^48^

#### Thermo analysis

**Fig. 8:**
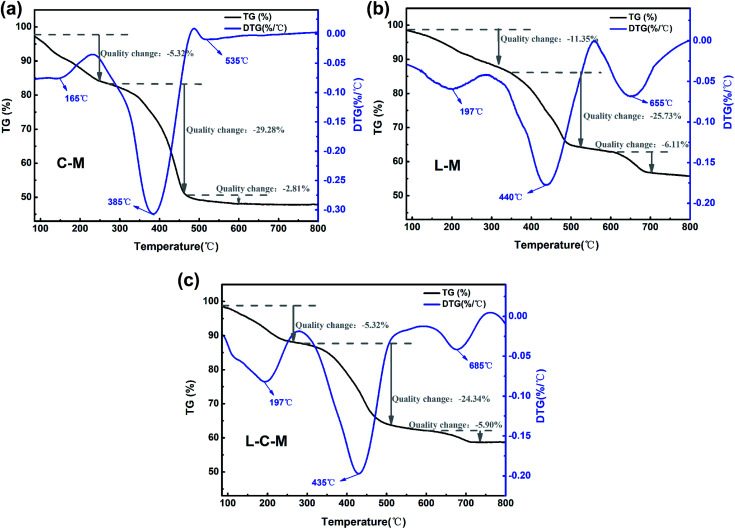
TG and DTG curves of the (a) C–M, (b) L–M and (c) L–C–M.

The TG curves and derivative TG (DTG) curves of the C–M, L–M and L–C–M materials are shown in Fig. 8. From the TG curve, it can be observed that the mass loss of all materials occurs in three stages. The first thermic event compares to the loss of mass around 160–200 °C, mainly owing to the liberation of physically adsorbed water on the outer surface of the catalyst. The second thermal event is in the temperature range of 380 °C to 450 °C, corresponding to the DTG curve. It is observed that there is a large weight loss peak at this stage, which is the removal of some chemically bound water in the catalyst, the dehydroxylation of the interlayer hydroxyl and the decomposition of carbonate.^29^ The third is at 530–660 °C, during which, the total mass changes the least, and gradually tends to thermal stability, which is mainly due to the release of carbon dioxide from carbonate during roasting, reflecting the existence of alkaline sites.^49^

However, the introduction of lanthanum metal moved the signal slightly towards a higher degradation temperature (DTG curves) (Fig. 8a–c). This result can be attributed to the influence of the dehydroxylation temperature of La_2_O_3_ on the particle size and crystallinity.^50^ Due to the change in crystal grains and size, the speed at which it reaches equilibrium is delayed because different crystal grains and sizes have different gas interference diffusion rates. These results are consistent with those obtained using the structure size of the material changes, and the amorphous property increases with the introduction of lanthanum. Therefore, all these factors may lead to an increase in the decomposition temperature of oxides.

#### Catalytic performance evaluation

**Fig. 9:**
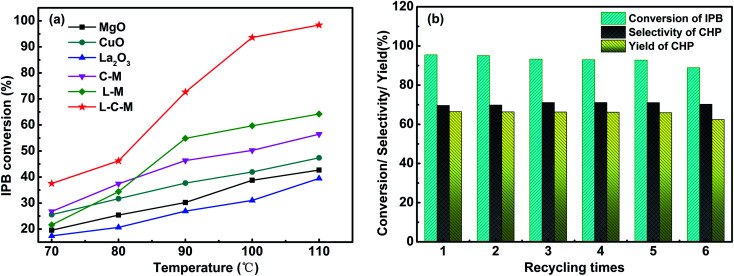
IPB conversion (a) and cyclic stability test results of L–C–M samples (b).

The catalytic activity test results of C–M, L–M, L–C–M, and single metal oxide for IPB total oxidation are shown in Fig. 9a. The catalytic activity of the composite metal oxide is better than that of single metal, especially after doping with rare earth metal lanthanum, its conversion rate is greatly improved. According to BET and SEM results, the doping of La makes the pore size distribution of catalyst crystals more dispersed, strengthens the interaction between Cu–Mg, produces more lattice defects, and promotes the adsorption of active oxygen on hydrogen atoms on isopropyl tertiary carbon more easily, thus showing better oxidation performance of IPB. The element ratio of the catalyst and the reaction conditions were also studied. As shown in Table 4, we investigated the elemental ratio of the catalyst and found that L_0.8_–C_0.03_–M_1_ has the best catalytic activity. When the amount of Cu was increased, although the conversion of IPB increased slightly, the selectivity to CHP decreased significantly. According to the literature, due to the strong redox performance of Cu, an increase in its content leads to poor dispersibility. The interaction force between La and Cu is obviously greater than the rate of oxygen overflowing to the carrier surface. It causes the rapid decomposition of CHP, and the content of alcohol and copper increases and the selectivity of CHP decreases. Therefore, it is impossible to continue to provide isopropyl radicals for branching reactions.^3^ So in the exploration of reaction conditions, too high a temperature or too fast oxygen flow rate will affect the state of provided free radicals, and then affect the yield of CHP. After single factor optimization (Fig. S1†), the conversion of IPB reaches 95.50%, the selectivity and yield of CHP reach 60.57% and 57.84%, respectively. The oxygen flow rate was 500 ml min^−1^ and 90 °C for 6 h from 2 wt% L_0.8_–C_0.03_–M_1_.

**Table 4:** SB sites, the molar ratio of O_β_ and catalytic oxidation activity of different composite oxide catalysts^a^

Samples	SB sites		O_β_/O_β_ + O_α_^b^ (%)	*C*_IPB_ c (%)	*S* (%)			*Y*_CHP_ e (%)
	Temperature (°C)	Quantity (cm^3^ per g STP)			CHP^d^	PP	AP	
C–M	670.25	2.32	0.65	53.26	56.59	34.69	8.43	30.14
L–M	633.92	30.14	0.67	67.50	58.90	39.56	0.35	39.76
*Co(BPZ)	—	—	—	34	84	15	1	28.56
*[MP_4_]Br	—	—	—	16.7	87.7	—	—	14.65
*Co-BTC	—	—	—	49	69	—	—	33.81
*NHPI/Cu(acac)_2_	—	—	—	68	44	45	10	29.92
L_0.8_–C_0.01_–M_1_	—	—	—	81.35	70.64	27.43	0.57	57.46
L_0.8_–C_0.03_–M_1_	672.43	46.60	0.71	95.50	60.57	36.23	1.75	57.84
L_0.8_–C_0.05_–M_1_	—	—	—	95.75	55.37	39.46	3.43	53.02
L_0.8_–C_0.07_–M_1_	—	—	—	96.23	50.39	43.12	5.85	48.49
L_0.8_–C_0.09_–M_1_	—	—	—	98.22	45.64	44.27	9.79	44.83

a Reaction conditions: raw material (IPB) = 20 ml, initiator (CHP) = 0.2 ml, composite oxide catalyst = 0.4 g, reaction temperature = 90 °C; time = 6 h; rate = 500 ml min^−1^.

b The surfer element molar ratio of O_β_ in O 1s XPS test.

c Conversion of IPB based on the DAD-HPLC results = moles of IPB reacted/[initial moles of IPB used] × 100.

d Selectivity of product calculated by total moles of CHP formed/total moles of IPB converted.

e Yield of CHP = conversion × selectivity.

Furthermore, based on the results of CO_2_-TPD and O 1s XPS and the data in Table 4, the L–C–M sample not only shows more SB sites for stimulating the reaction activity but also has more O_β_. As we know, O_β_ performs a significant part in the oxidation reaction system, that can effectively utilize the lattice defects in catalytic molecules to adsorb and activate quickly and can transfer and replenish automatically, thus accelerating the circulation of the reaction. It is precise because of its high oxygen mobility that effectively inhibits the coking of the catalyst. Notably, rare earth lanthanum itself has good oxygen storage and release capacity, thus effectively enhances the catalytic life of the catalyst. In this study, the recovered L–C–M sample was washed with ethanol (specific operations are added in the ESI†), and then the IPB catalytic oxidation experiment was repeated many times. As shown in Fig. 9b, the catalytic activity did not change significantly, indicating that it has good stability and catalytic life.

**Fig. 10:**
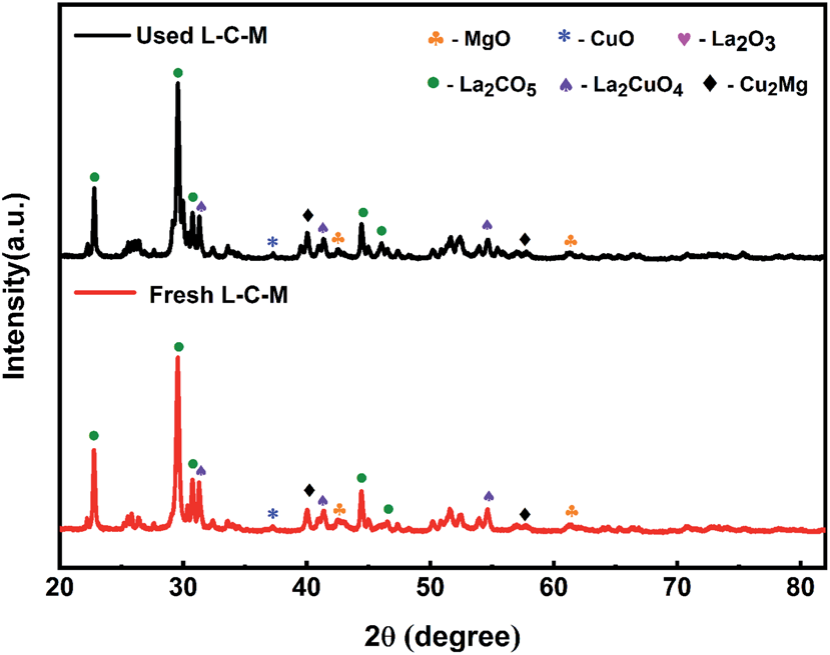
XRD patterns profiles of fresh and used samples.

The samples were characterized and compared using XRD after several stability tests of the recovery cycle. As displayed in Fig. 10, the diffraction patterns on fresh samples and used samples have no obvious changes, which indicates that each phase and particle size about the samples are not affected after being used many times.

#### Possible promotional mechanism analysis

**Fig. 11:**
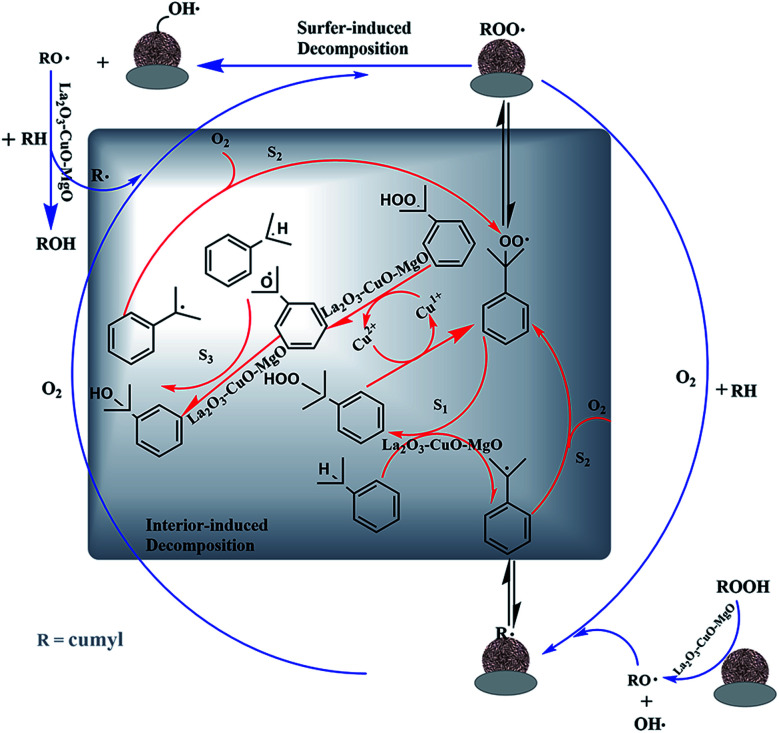
Catalytic mechanism of cumene free radical chain oxidation.

Summarizing the results obtained from this study and works from literature, the possible reaction mechanism of the oxidation of IPB on the L–C–M catalyst is presented in Fig. 11. The peroxidation of cumene can be described as a typical free radical chain reaction.^13,51^ In most cases, the process can be regarded as a predetermined automatic oxidation process for reactions (1)–(5).^52,53^ Its catalytic peroxidation process on L–C–M involves the combination of free radical reactions on the surface of and inside of particles.

Initiation:

1 ROOH → RO˙ + ˙OH

2 RO˙ + RH → ROH + R˙

Propagation:

3 R˙ + O_2_ → ROO˙

4 ROO˙ + HR → ROOH + R˙

Termination:

5 2ROO˙ → non-radical products

The initiation (eqn (1) and (2)), propagation (eqn (3) and (4)), and termination (eqn (5)) are typical steps that occur in free radical chain reactions.

The particle surface involves rapid adsorption of CHP (initiator formed and added during the entire reaction). Because of the strong interaction between La_2_O_3_–CuO and Cu^2+^ ions, well-dispersed CuO species are distributed around its site (–La^3+^–Ov–Cu^2+^–O–La^3+^–) and form more oxygen vacancies (Ov) to become the adsorption and activation sites of CHP, which are then decomposed into RO˙ and HO˙.^54^ This process is also well reflected in the internal reaction, especially for the part that induces the activation of IPB. Cu^2+^ itself has the effect of initiating a chain reaction to generate RO˙ radicals, thereby accelerating the effective progress of the reaction.

The alkaline earth metal magnesium oxide has a special effect on the activation of IPB. The negatively charged lattice oxygen provided by the alkali center on the surface of the alkaline earth metal catalyst is chemically adsorbing the hydrogen atom from the isopropyl tertiary carbon, thus weakening the C–H bond on the isopropyl tertiary carbon. According to the results of O 1s XPS, more lattice and active oxygen species are generated in the L–C–M catalyst, making more O^2−^ interact with the hydrogen bond of IPB tertiary carbon. As a result, a large number of surface oxygen vacancies are used for the adsorption–activation of oxygen molecules, which improves the mobility of oxygen and accelerates the overall conversion rate.^55^ In the reaction process, the dissociation enthalpy of the O–H bond is 104 kcal mol^−1^ in benzol RO–H and 88 kcal mol^−1^ in CHP ROO–H.^56^ The rate of extracting hydrogen atoms (S2) from IPB molecules by RO˙ free radicals is much faster than that of hydrogen abstraction reaction (S1) between ROO˙ and IPB.^3^ The formation of CHP and R˙ (S2) becomes the control step of the whole radical chain reaction. Improving transformation capability is an efficient way to effectively stimulate the tertiary carbon–hydrogen bond for IPB. Therefore, improvements in oxygen adsorption–activation and L–C–M interaction both contribute to the superior catalytic performance in IPB total oxidation.

## Experimental section

### Materials

The catalytic oxidation of cumene was implemented on three catalysts *viz.*, CuO–MgO (C–M), La_2_O_3_–MgO (L–M), and La_2_O_3_–CuO–MgO (L–C–M). All the chemicals used to prepare the catalyst were of AR grade. Cumene and CHP were purchased from Shanghai Aladdin Biochemical Technology Co., Ltd. Magnesium nitrate hexahydrate, copper nitrate trihydrate, lanthanum nitrate hexahydrate, and anhydrous sodium carbonate were provided by Sinopharm Chemical Reagent Co., Ltd., oxygen was procured from Nanjing Tianze Gas Co., Ltd.

### Preparation of CuO–MgO

C–M was prepared by dropping a 1 mol l^−1^ of Na_2_CO_3_ aqueous solution into 100 ml stirred aqueous solution of Cu(NO_3_)_2_·3H_2_O and Mg(NO_3_)_2_·6H_2_O weighed under different molar ratios as *n*(Cu) : *n*(Mg) under constant churning. The precipitate was washed to a pH of 7. Filtered and dried in an oven at 373 °C for 12 hours. The dried sample was next calcined in air at 873 K for 6 h to obtain C–M.

### Preparation of La_2_O_3_–MgO

L–M was prepared by dropping a 1 mol l^−1^ Na_2_CO_3_ aqueous solution into 100 ml of a blended aqueous solution of La(NO_3_)_3_·6H_2_O and Mg(NO_3_)_2_·6H_2_O weighed in line with different molar ratios of *n*(La) : *n*(Mg) under continual agitating. The precipitate was washed until the pH of the washing was 7, filtered, and the cake was dried in the oven at 373 °C for 12 hours. The dried sample was later calcined in air at 973 K for 6 h to acquire L–M.

### Preparation of La_2_O_3_–CuO–MgO

L–C–M was prepared by dropping 1 mol l^−1^ of Na_2_CO_3_ aqueous solution into 100 ml of a mixed aqueous solution of Cu (NO_3_)_2_·3H_2_O, La(NO_3_)_3_·6H_2_O and Mg(NO_3_)_2_·6H_2_O weighed according to different molar ratios of *n*(La) : *n*(Cu) : *n*(Mg) under continuous stirring. The precipitate was washed until the pH of the supernatant was 7, then filtered and the cake was dried in the oven at 373 °C for 12 hours. The dried sample was calcined in air at a temperature of 873 K for 6 h to obtain L–C–M.

### Catalysts characterization

X-ray powder diffraction (XRD) patterns were collected on a Rigaku XRD-6000 diffraction instrument equipped with Cu Kα radiation (*λ* = 1.5406 Å) (Shimadzu, Kyoto, Japan). The microstructures of the materials were characterized using a scanning electron microscope (SEM, Hitachi-S4800, Tokyo, Japan). Energy-dispersive (EDS) study was performed using an EX 250 spectrophotometer (Horiba, Kyoto, Japan). Fourier transform infrared spectroscopy (FT-IR) was performed on TNEXUS-670 (Thermo Nicolet Corporation, USA). The surface area (BET) measurements by nitrogen adsorption at 77 K were determined by ASAP 2020. Before the measurement, samples were heated in a vacuum at 353 K. CO_2_-TPD was conducted using an AutoChem II 2920 apparatus to survey the total alkalinity and alkali strength. X-ray photoelectron spectroscopy (XPS) examination was performed on an ESCALAB 250Xi instrument (Thermo, USA). TG-DTA was conducted from 100 to 800 °C at a heating rate of 10 °C min^−1^ and 100 ml min^−1^ of airflow on a thermobalance (PerkinElmer, USA).

### Oxidation on the catalyst

As a representative reaction, liquid-phase oxidation of cumene was performed in a circular-bottom flask equipped with a condenser. Raw materials cumene, solid base catalyst (2%), and initiator CHP (1%) were added in succession into the flask. The reaction was conducted with continuous stirring at a certain time and temperature. After the reaction, the products were analyzed using Agilent High-Performance Liquid Chromatography (Model 1260). The product identification was performed by diluting the standard sample with methanol and injecting it into the high-performance liquid chromatography. The catalyst was reclaimed through centrifugation and washed thoroughly with ethanol for reuse.

## Conclusions

The following are the major conclusions of the work: (1) the results show that the IPB oxidation reaction catalyzed on the L–C–M catalyst showed higher activity and yield. Which provides a potential scheme for the related research due to its low cost, convenient post-treatment, and being environment-friendly. (2) The higher the dispersion of copper and magnesium particles, the stronger is the synergism between lanthanum and copper and magnesium, which seems to be the reason for the higher activity of the L–C–M catalyst, according to the characterization results of XRD, SEM, CO_2_-TPR, and XPS. Its role is to promote the transfer of electrons between ions, induces the formation of more oxygen vacancies, thereby improving the volume of chemically adsorbed oxygen on the catalyst surface and improve the oxidation performance. (3) The cyclic stability test of L–C–M shows that its high oxygen mobility inhibits the coking of the catalyst, so it maintains a good crystal structure and stability, as evidenced from O 1s XPS analysis.

## Conflicts of interest

There are no conflicts to declare.

## Supplementary Material

RA-011-D1RA00176K-s001
