# Supplementary material for: A new highly active La2O3–CuO–MgO catalyst for the synthesis of cumyl peroxide by catalytic oxidation
Source: RSC Adv. 2021 Mar 30;11(21):12532–42. doi: 10.1039/d1ra00176k (PMC8696952; doi:10.1039/d1ra00176k)
Supplement: RA-011-D1RA00176K-s001 [file RA-011-D1RA00176K-s001.pdf]

Electronic Supplementary Material (ESI) for RSC Advances.
This journal is © The Royal Society of Chemistry 2021

Supplementary Information

A new highly active La_2O_3-CuO-MgO catalyst for the synthesis of cumyl peroxide by catalytic oxidation

HanShuang Liu, [a] KaiJun Wang, [a] XiaoYan Cao,[a] JiaXin Su,[a] and Zhenggui Gu*[a]

Jiangsu Provincial Key Laboratory of Materials Cycling and Pollution Control, Nanjing Normal Univesity, Nanjing, Jiangsu 210023 China.

*Corresponding authors: Zhenggui Gu

Email:07160@njnu.edu.cn

Supporting Text

Catalyst recovery operation: Firstly, the catalyst after each reaction is preliminarily separated from the product CHP by vacuum filtration. Adding 50ml ethanol, stirring for 10min under magnetic stirring, and filtering, and repeating the operation twice to remove adhered organic compounds. Then wash and filter with deionized water, and dry the filter cake at 100℃ for 12 hours to repeat the experiment.

Supporting Figure

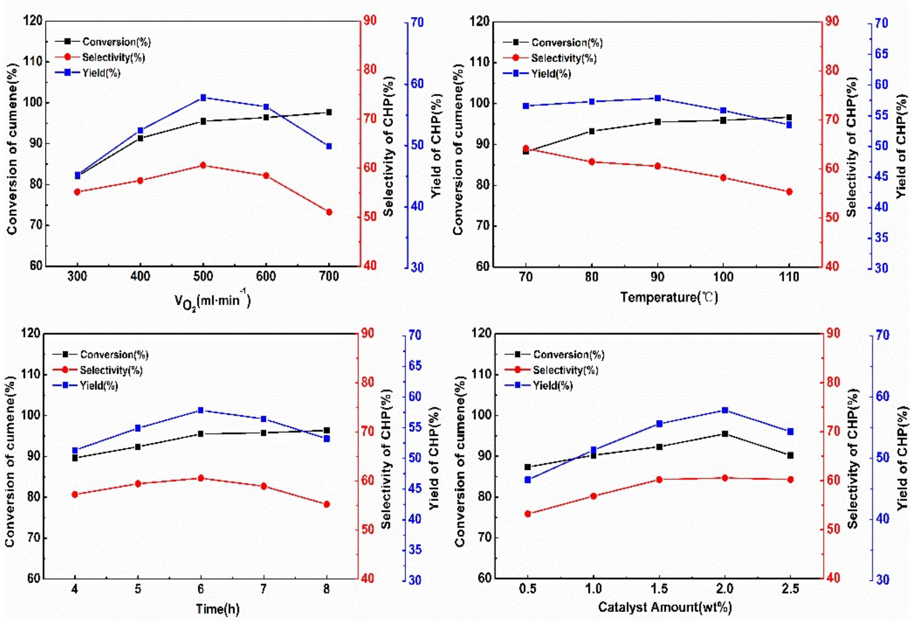

Figure S1 Single factor optimization of the reaction.
